# Mechanism of Reactive Oxygen Species-Guided Immune Responses in Gouty Arthritis and Potential Therapeutic Targets

**DOI:** 10.3390/biom14080978

**Published:** 2024-08-09

**Authors:** Sai Zhang, Daocheng Li, Mingyuan Fan, Jiushu Yuan, Chunguang Xie, Haipo Yuan, Hongyan Xie, Hong Gao

**Affiliations:** 1 Hospital of Chengdu University of Traditional Chinese Medicine, Chengdu 610075, China; zhangsai@stu.cdutcm.edu.cn (S.Z.);; 2 TCM Regulating Metabolic Diseases Key Laboratory of Sichuan Province, Chengdu 610072, China; 3 Department of Endocrinology, Hospital of Chengdu University of Traditional Chinese Medicine, Chengdu 610032, China

**Keywords:** gouty arthritis, ROS, oxidative stress, immune responses, treatment

## Abstract

Gouty arthritis (GA) is an inflammatory disease caused by monosodium urate (MSU) crystals deposited in the joint tissues causing severe pain. The disease can recur frequently and tends to form tophus in the joints. Current therapeutic drugs for the acute phase of GA have many side effects and limitations, are unable to prevent recurrent GA attacks and tophus formation, and overall efficacy is unsatisfactory. Therefore, we need to advance research on the microscopic mechanism of GA and seek safer and more effective drugs through relevant targets to block the GA disease process. Current research shows that the pathogenesis of GA is closely related to NLRP3 inflammation, oxidative stress, MAPK, NET, autophagy, and Ferroptosis. However, after synthesizing and sorting out the above mechanisms, it is found that the presence of ROS is throughout almost the entire spectrum of micro-mechanisms of the gout disease process, which combines multiple immune responses to form a large network diagram of complex and tight connections involved in the GA disease process. Current studies have shown that inflammation, oxidative stress, cell necrosis, and pathological signs of GA in GA joint tissues can be effectively suppressed by modulating ROS network-related targets. In this article, on the one hand, we investigated the generative mechanism of ROS network generation and its association with GA. On the other hand, we explored the potential of related targets for the treatment of gout and the prevention of tophus formation, which can provide effective reference ideas for the development of highly effective drugs for the treatment of GA.

## 1. Introduction

Gouty arthritis (GA) is an inflammatory disease caused by the deposition of sodium urate (MSU) crystals in the joints or extra-articular tissues triggering severe pain, which is mostly self-limiting but frequently recurring, troubling a wide range of patient populations [1]. According to the latest research study, as of 2020, the prevalence of GA in all countries around the world is distributed between <1% and 6%, with an annual incidence of 0.58–2.89 per 1000, and shows a higher trend of prevalence in males than in females with increasing age [2]. The most important cause of GA episodes is the formation of hyperuricemia. In the physiological state, uric acid (UA) is the end product formed after the metabolism of purines in the human body in the liver and intestines, of which two-thirds are metabolized and excreted by the kidneys, and the remaining third through the gastrointestinal tract [3]. However, an increase in UA production or a decrease in excretion can lead to the excessive accumulation of UA. When the amount of UA accumulated in the blood exceeds 404 μmol/liter (6.8 mg/dL), it accelerates the formation of MSU crystals deposited in the joints, and can induce an acute inflammatory response leading to GA attacks. Over time, these crystals accumulate, and can even lead to the formation of tophus [4]. In addition, it has been found that even if GA is in remission at the stage of hyperuricemia, it is still very harmful to the human body. The clinical symptoms of hyperuricemia are not obvious and are often overlooked, but the long-term existence of “high uric acid” deposition in a variety of tissues seriously jeopardizes the development of chronic kidney disease, cardiovascular disease, diabetes, and malignant tumors by inducing inflammation, endothelial dysfunction, oxidative stress (OS), insulin resistance, vascular smooth muscle cell proliferation, and renin angiotensin activation [5]. Therefore, alleviating acute GA attacks, lowering blood MSU crystals, and preventing the formation of tophus are major issues facing GA patients today.

## 2. Mechanisms of GA Onset and Its Link to ROS

With regard to the mechanism underlying the formation of the acute exacerbation phase of GA, it is widely believed to be closely related to the inflammatory cascade response triggered by MSU crystals acting as endogenous signals to activate NOD-like receptor thermal protein domain-associated protein 3 (NLRP3) inflammatory vesicles in phagocytes [6,7,8]. However, current research suggests that in addition to the NLRP3 inflammatory pathway, the production of reactive oxygen species (ROS) is another important cause of GA attacks [9,10]. A synthesis of several studies found that OS markers were significantly and positively correlated with MSU crystal concentration, GA attack frequency, and symptom severity [11,12,13,14]. Further studies have shown that xanthine oxidase (XO) [15], nicotinamide adenine dinucleotide phosphate oxidase (NADPH oxidase) (NOX) [16], and signals such as phosphoinositide 3-kinase (PI3K)/protein kinase B (AKT) [17], mitogen-activated protein kinase (MAPK), and NET, which are closely related to ROS production and metabolism, are abnormally active in hyperuricemia or GA development. XO is a key enzyme in UA production, thus suggesting that hyperuricemia is closely related to ROS production. It is important to note that ROS are not only produced concomitantly during UA formation, but also through the UA stimulation of NOX activity, the reduction in NO metabolism by decreasing endothelial nitric oxide synthase activity, and the enhancement of the affinity of arginase for L-arginine to promote ROS production [18,19]. Moreover, UA can significantly induce chondrocyte OS in a time- and concentration-dependent manner, resulting in chondrocyte dysfunction, cartilage damage, and the formation of chronic cartilage erosion, which is one of the most important prerequisites for triggering GA [20]. In addition, MSU crystals formed from UA can also induce large amounts of ROS generation and cause cell death by increasing NOX activity, promoting lysosomal damage, and inducing mitochondrial membrane dysfunction [21,22,23]. Recently, it has been shown that ROS production is also cross-talked and interdependent with NLRP3 inflammatory signaling activated by MSU crystals. ROS can specifically activate the NLRP3 inflammasome; conversely, the activated NLRP3 inflammasome can release ROS by inducing an inflammatory response and recruiting neutrophils and macrophages to play the role of immunity, which can further contribute to damage to the soft tissues of the joints [24]. In the last decade, with the gradual enrichment of the mechanism of neutrophil extracellular traps (NETs), it was found that it occupies an important position in the formation of tophus during acute exacerbation, remission, and late tophus formation in GA [25,26,27]. NETs can both relieve the acute inflammatory response and participate in the formation of tophus. One of the necessary conditions for the formation of NETs is precisely the production of ROS, and their activation, in turn, causes the release of more ROS [28]. Under the environment of hyperuricemia, when MSU crystals are formed and induced to produce a large amount of ROS, they directly oxidize cell membrane lipids to produce the lipid peroxides malondialdehyde (MDA), and also oxidatively damage intracellular proteins, various types of enzymes, and cytosolic DNA. Furthermore, ROS can also act as a signaling molecule to induce apoptotic pathways, counteract autophagy, promote inflammatory responses, and impair mitochondrial function, among other mechanisms, to disrupt the intracellular environment, leading to cellular damage or death, and ultimately damaging the structural function of joint tissues [29,30]. In the acute phase of GA, the ROS production induced by MSU crystals activates transient Receptor Potential Ankyrin 1 (TRPA1) in sensory neurons as well as transient receptor potential vanilloid-1 (TRPV1), which are expressed by a subpopulation of peptidergic damage receptors that can drive GA inflammation and pain [31]. In summary, upstream and downstream ROS form a huge network that participates in the development of GA from multiple directions, and it is urgent to clarify the mechanism of the ROS network in GA and the related therapeutic targets.

This paper provides a detailed compendium of the mechanisms of ROS production, metabolism, and signaling, and outlines the pathological relationship between them and GA. Based on the understanding of oxidative stress damaging, body antioxidant defense function, and the mechanism of the ROS network regulating inflammation, autophagy, and NET, we summarize and analyze the targets that may be utilized in the treatment of GA using the ROS network, and collect the research data on the application of the ROS network mechanism to GA in the past 10 years in this paper. The efforts we made are expected to break through the existing anti-GA therapeutic means and to provide effective reference ideas for efficient drugs targeting the ROS network to treat GA.

## 3. ROS and OS Generation Mechanism

**Figure 1:**
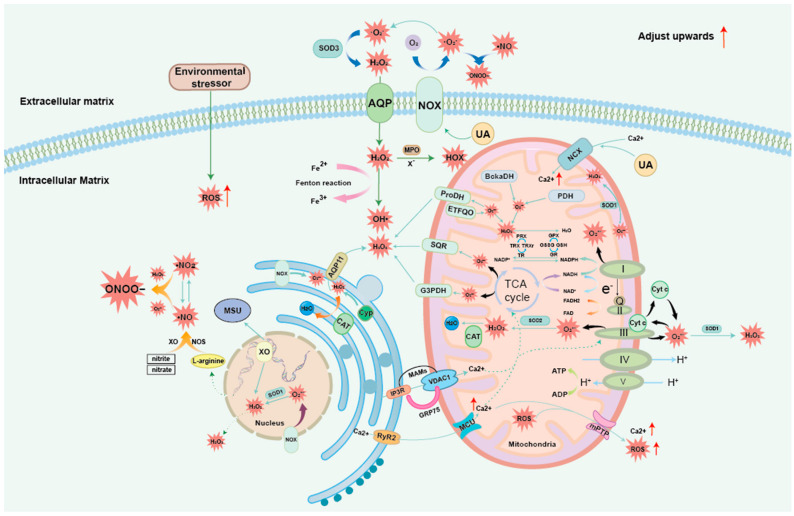
ROS generation mechanism in GA: The pathway of ROS production in the organism is broadly categorized into exogenous stimulation and endogenous activation, in which endogenous activation of ROS production scenarios mainly include the mitochondria, endoplasmic reticulum, and the nucleus, and a variety of metabolic disorders can contribute to the abnormal accumulation of ROS, in which uric acid is one of the triggers of ROS production, and the production of excess ROS is an important prerequisite for the disease process of GA. ADP: Adenosine Diphosphate; ATP: Adenosine Triphosphate; AQP11: Aquaporin11; BckaDH: branched-chain ketoacid dehydrogenase; CAT: catalase; Cyp: cytochrome P450; Cyt c: Cytochrome c; ETFQO: electron transfer flavoprotein oxidoreductase; FAD: flavin adenine dinucleotide; FADH2: flavin adenine dinucleotide; G3PDH: dehydrogenase; GPX: glutathione peroxidase; GR: glutathione reductase; GRP75: glucose-regulated chaperone protein 75; GSH: glutathione; GSSG: oxidized glutathione; H_2_O_2_: hydrogen peroxide; IP3R: inositol 1,4,5-trisphosphate receptors; MCU: Mitochondrial Calcium Uniporter; MPO: Myeloperoxidase; mPTP: mitochondrial permeability transition pore; MSU: monosodium urate; NAD^+^: Nicotinamide adenine dinucleotide; NADH: ubiquinone oxidoreductase; NADPH: nicotinamide adenine dinucleotide phosphate oxidase; •NO: nitric oxide; •NO_2_: nitrogen dioxide; NOS: nitric oxide synthase; NOX: NADPH oxidase; O_2_^•−^: superoxide anion; PDH: pyruvate dehydrogenase; ProDH: proline dehydrogenase; PRX: peroxiredoxin; ROS: reactive oxygen species; RyR2: ryanodine receptors type 2; SOD: superoxide-dismutase; SQR: succinate: quinone reductase; TRX: Thioredoxin; TrxR (TR): Thioredoxin reductase; UA: Uric acid; VDAC1: voltage-dependent anion channel 1; XO: xanthine oxidase.

OS is a state in which a large amount of ROS is generated in the organism due to the triggering of certain signals, and then its strong oxidative properties are utilized to cause an imbalance between the oxidative and antioxidative functions in an organism, causing multiple damages to tissue cells [32]. In fact, ROS does not refer to one substance in particular but is a general term for a class of substances with strong oxidizing properties. Investigative studies have found that the types of ROS include superoxide anion free radical, singlet oxygen, hydroxyl radical, ozone, hydrogen peroxide, hypochlorous acid, hypoiodous acid, hypobromous acid, and organic peroxides, etc. [33]. There are different opinions on the causes of ROS generation, but they are broadly categorized into two pathways, namely exogenous stimuli and endogenous oxygen metabolism [34,35]. Figure 1 summarizes the mechanism of ROS generation and metabolism.

### 3.1. Exogenous ROS

Exogenous stimuli can be caused by environmental stressors (e.g., environmental pollutants, drugs such as cyclosporin or gentamicin, ionizing or X-ray radiation, heavy metals such as Cd or Pb, ultraviolet light, certain chemical solvents, cigarette smoke, cooking smoke, and alcohol). When the body metabolizes these exogenous compounds, it produces a variety of by-products, including large amounts of ROS [36].

### 3.2. Mitochondrial ROS

Endogenous oxygen metabolism in the organism can be generated through several pathways, a large group of which originates from leakage from the mitochondrial respiratory chain [37]. The amount of oxygen consumed by mitochondria in an organism reaches 80–95% and generates 95% of the cellular energy through the oxidative phosphorylation (OXPHOS)-coupled tricarboxylic acid (TCA) cycle and the mitochondrial electron transport chain (ETC). During OXPHOS, mitochondrial ROS is accompanied by mitochondrial ETC production [38]. According to the current study, the ROS produced by mitochondria under physiological conditions account for 2% of the total mitochondrial oxygen consumption, whereas in pathological conditions, this percentage can reach up to 11% [39]. Playing a major role in ETC ROS production are four complexes located on the inner mitochondrial membrane, namely, complex I: ubiquinone oxidoreductase (NADH), complex II: succinate dehydrogenase, complex III: cytochrome c reductase, and complex IV: cytochrome C oxidase [40]. A series of redox reactions occur during the sequential transfer of electrons in complexes I–IV. Complex I can oxidize to form nicotinamide adenine dinucleotide (NAD^+^) and H^+^ after transferring electrons to coenzyme Q. Complex II can form flavin adenine dinucleotide (FAD) after transferring reduced flavin adenine dinucleotide (FADH2) electrons to coenzyme Q. The coenzyme Q that collects the electrons transfers the electrons to complex III, which can then process oxygen to obtain superoxide anion (O_2_^•−^), and O_2_^•−^ generates H_2_O_2_ under the action of mitochondrial manganese superoxide dismutase (MnSOD). Complex IV has two roles. One is to act as a proton channel to pump H^+^ from the mitochondrial matrix to the endomembrane to pump it out, creating an H^+^ concentration gradient that provides the basis for the conversion of adenosine diphosphate (ADP) to adenosine triphosphate (ATP). The other role is to combine some of the H^+^ with transferred electrons and oxygen to form H_2_O [41,42,43]. In addition, intra-mitochondrial superoxide can also form H_2_O_2_ catalyzed by a variety of enzymes, including branched-chain ketoacid dehydrogenase (BckaDH), pyruvate dehydrogenase (PDH), proline dehydrogenase (ProDH), 2-oxoglutarate dehydrogenase (ODH), superoxide-dismutase-2 (SOD2), electron transfer flavoprotein oxidoreductase (ETFQO), glycerol-3-phosphate dehydrogenase (G3P), glycerol-3-phosphate dehydrogenase (G3PDH), and succinate/quinone reductase (SQR) [44]. In addition to the aforementioned ROS production by ETCs in mitochondria, most unexpectedly, ROS themselves can induce ROS production. It has been noted that ROS can separate cytochrome c from the inner mitochondrial membrane (IMM) cardiolipin (CL), preventing it from transferring electrons from complex III to complex IV, and, thereafter, electrons accumulate on respiratory complexes I and III, further increasing their ROS production [41,45].

### 3.3. Common ROS: H_2_O_2_

Among the many types of ROS, H_2_O_2_, as the most common ROS in the organism, is produced and metabolized in a variety of ways in the cell. Firstly, in addition to the above-mentioned mitochondria that can produce a large amount of H_2_O_2_, folding proteins in the endoplasmic reticulum’s inner lumen and the process of forming disulfide bonds are also a major source of H_2_O_2_ production. The endoplasmic reticulum also contains enzymes of the cytochrome P450 (Cyp) family, which are involved in the synthesis of steroids and the oxidative detoxification process of several exogenous substances, and these systems can generate considerable O_2_^•−^ and H_2_O_2_ fluxes and efflux through the endoplasmic reticulum’s proprietary Aquaporin11 (AQP11) channel [46]. Secondly, some H_2_O_2_ is produced intracellularly, in mitochondria and in the nucleus, such as SOD1-SOD3, which can produce H_2_O_2_ in a targeted manner; however, this process is often likened to the first line of defense against oxidative damage as it consumes more oxidative O_2_^•−^ [47,48,49]. Another class of enzymes, XO, can also be directed to produce H_2_O_2_ and O_2_^•−^. This process occurs when XO catalyzes hypoxanthine and xanthine, and the second important product formed by the reaction is UA, which also serves as one of the prerequisites for the highly OS state of GA tissues and is an important bridge connecting GA to ROS [50]. The H_2_O_2_ formed during the above process can be directly decomposed into H_2_O under the action of catalase (CAT) [51]. In addition, the presence of O_2_^•−^ will cause the release of Fe^2+^ from iron sulfur proteins. Under the action of metal ions, such as Fe^2+^ and Cu^2+^, H_2_O_2_ can be decomposed into the more oxidizing OH^−^, which is often referred to as the Fenton reaction and has a very strong destructive effect on the proteins and DNA in the cells [52]. In addition to the above, H_2_O_2_ can also be reduced to H_2_O under the action of reduced glutathione (GSH); however, this reaction is dependent on nicotinamide adenine dinucleotide phosphate (NADPH) with oxidized glutathione (GSSG) in the presence of the formation of GSH and glutathione peroxidase (GPX) substrates catalyzed by glutathione reductase (GR). H_2_O_2_ can also be decomposed into H_2_O through the peroxiredoxin (PRX)/thioredoxin (TRX) system and then loses its oxidizing properties [53,54]. It has also been reported that in the presence of myeloperoxidase (MPO), X- (e.g., Cl^−^, Br^−^ or SCN^−^) can react with H_2_O_2_ to form hypohalous acid (HOX) and H_2_O [55]. For example, Cl^−^, as a substrate for MPO-mediated catalytic reactions, leads to the formation of hypochlorous acid (HOCl), which is 50 times more oxidizing than H_2_O_2_ [56].

### 3.4. Nitrogen Oxides

Another class of ROS is the formation of nitrogen oxides, including nitric oxide (•NO), nitrogen dioxide (•NO_2_), and peroxynitrite (ONOO^−^) [38,57]. •NO is usually produced when nitric oxide synthase (NOS) oxidizes arginine, and can also be produced when nitrite and nitrate are catalyzed by different types of enzymes (e.g., hemoglobins, molybdo-flavoproteins, mitochondrial proteins and Cyp). •NO_2_ and •NO have the ability to interconvert with each other, and both •NO and •NO_2_ can be converted to ONOO^−^ by different reactions. Among them, •NO depends on the participation of O_2_^•−^, while •NO_2_ depends on the reaction with H_2_O_2_, and the generated ONOO^−^ is another strong oxidant in the organism after OH^−^ and HOCl, which can directly react with DNA, proteins, and lipids and play a destructive role [58].

### 3.5. Endoplasmic Reticulum-Mitochondria ROS

It should also be noted that the Ca^2+^-directed endoplasmic reticulum (ER)-mitochondrial coupling system is another important pathway to stimulate ROS production [59]. Mitochondria-associated membranes (MAMs) are functional structures connecting mitochondria to the ER and allow mitochondria to tightly organize around the ER membrane. There are three channels between mitochondria and the ER: the inositol 1,4,5-trisphosphate receptors (IP3R) at the ER terminus, voltage-dependent anion channel 1 (VDAC1) at the mitochondrial terminus, and the connecting cytosolic glucose-regulated chaperone protein 75 (GRP75), which, together, play a role in regulating Ca^2+^ circulation from the ER to the mitochondria [60,61,62]. Alternatively, Ca^2+^ can also flow out of the ER via ryanodine receptors type 2 (RyR2) and subsequently enter the mitochondria via the Mitochondrial Calcium Uniporter (MCU) and further stimulate the ETC activity, thus inducing more ROS production [63,64]. It was found that mitochondrial Ca^2+^ not only exacerbates ROS production, but also synergizes with O_2_^•−^ to promote the release of ROS to the extramitochondria [65]. When Ca^2+^ reaches a certain concentration in mitochondria, it induces the mitochondrial permeability transition pore (mPTP) supramolecular entity channel assemblies and opens at the IMM and the outer mitochondrial membrane (OMM), triggering the phenomenon of mitochondrial permeability transition (mPT). It allows up to 1.5 kDa of ions and solutes to move in and out of the mitochondria, including ROS and proteins. However, ROS entering the cytoplasm will generate chain reactions with other mPTPs on the mitochondrial surface that have not gone through the mPT by lowering the mPTP opening threshold, leading to the influx of more ROS into the cell, creating a vicious cycle that ultimately leads to destructive oxidative stress [66]. It is also worth mentioning that high concentrations of UA stimulation can also stimulate Ca^2+^ overload through massive influx into the mitochondria, resulting in the generation of large amounts of ROS. However, such phenomena are mediated through another cation transporter protein on the mitochondria called a sodium calcium exchanger (NCX) [67].

## 4. Mechanisms of ROS-Guided Immune Responses in the GA Environment

**Figure 2:**
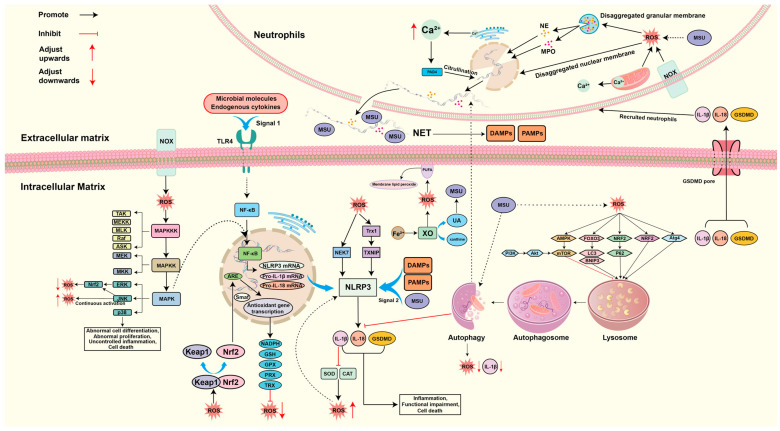
ROS network formation mechanism in GA: ROS not only contribute to tissue cell damage through the oxidative stress process in the gout disease process, but also act as signaling molecules to activate inflammation, autophagy, NET, and Ferroptosis to cause tissue cell damage in multiple ways, and each pathway interacts with each other, ultimately forming a large network diagram involved in the disease process of GA. AKT: protein kinase B; AMPK: Adenosine 5′-monophosphate (AMP)-activated protein kinase; Atg4: Autophagy protein 4; ARE: Antioxidant Response Element; ASK/Raf/MLK/MEKK/TAK: A member of the MAPKKK family; BNIP3: BCL2/adenovirus E1B 19 kDa interacting protein 3; CAT: catalase; DAMP: damage-associated molecular pattern; ERK/JNK/p38: A member of the MAPK family; FOXO3: Forkhead Box Protein O3; GPX: glutathione peroxidase; GSDMD: gasdermin D; GSH: glutathione; IL-1β/18: interleukin-1β/18; Keap1: Kelch-like ECH-associated protein 1; LC-3: microtubule-associated protein light chain 3; MAPK: mitogen-activated protein kinase; MAPKK: MAP kinase kinase; MAPKKK: MAP kinase kinase kinase; MEK/MKK: A member of the MAPKK family; MPO: Myeloperoxidase; MSU: monosodium urate; mTOR: mammalian target of rapamycin; NADPH: nicotinamide adenine dinucleotide phosphate oxidase; NCX: Sodium Calcium Exchanger; NE: neutrophil elastase; NEK7: NIMA-related kinase 7; NET: neutrophil extracellular traps; NF-κB: nuclear factor kappa-B; NOX: NADPH oxidase; NLRP3: NOD-like receptor thermal protein domain-associated protein 3; Nrf2: Nuclear factor erythroid2-related factor 2; P62: Sequestosome 1; PAD4: peptidylarginine deiminase 4; PAMP: pathogen-associated molecular pattern; PI3K: phosphoinositide 3-kinase; PRX: peroxiredoxin; ROS: reactive oxygen species; sMaf: small musculoaponeurotic fibrosarcoma; SOD: superoxide-dismutase; TLR4: Toll-like receptor 4; TRX: Thioredoxin; TXNIP: thioredoxin interacting protein; UA: Uric acid; XO: xanthine oxidase.

As mentioned earlier, ROS can be produced through multiple pathways in the GA environment. When high concentrations of ROS accumulate in tissue cells, they will directly act on and cause damage to intracellular proteins, DNA, lipid membranes, and other basic structures, inducing the onset of GA, whereas low concentrations of ROS will act as a second messenger to participate in the modulation of multiple immune responses and form a huge ROS network, including Nuclear factor erythroid2-related factor 2 (Nrf2)-Kelch-like ECH-associated protein 1 (Keap1), ROS-autophagy to alleviate OS and inflammation, or ROS-nuclear factor kappa-B (NF-κB)-NLRP3 inflammation, ROS-MAPK, ROS-NET, ROS-Ferroptosis to activate inflammation, apoptosis, and other pathways to cause cellular malfunction or even death [68,69]. Figure 2 summarizes the crosstalk mechanism of the ROS network and its role in GA.

### 4.1. ROS-NF-κB-NLRP3 Inflammation

**Figure 3:**
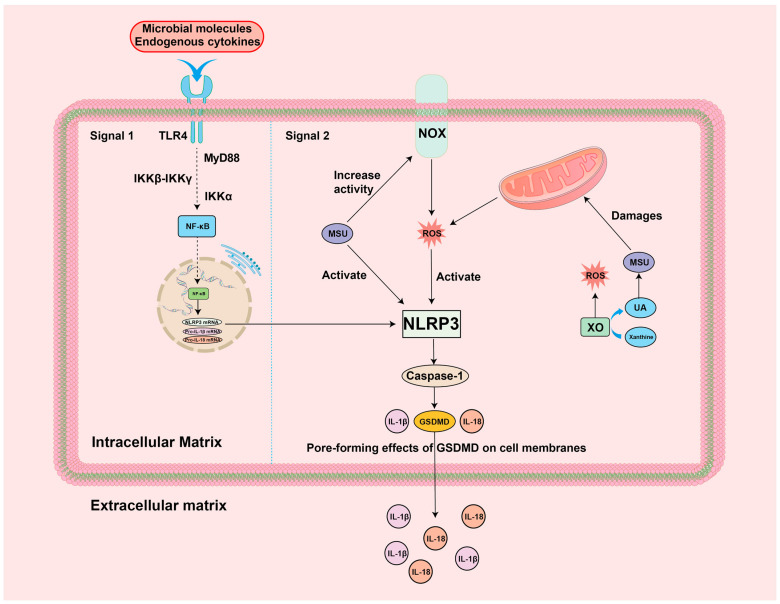
Mechanism of ROS-NLRP3 inflammation in GA environment: GSDMD: gasdermin D; IKK: Inhibitor of kappa B kinase; IL-1β/18: interleukin-1β/18; MSU: monosodium urate; MyD88: Myeloid differentiation primary response 88; NF-κB: nuclear factor kappa-B; NOX: NADPH oxidase; NLRP3: NOD-like receptor thermal protein domain-associated protein 3; ROS: reactive oxygen species; TLR4: Toll-like receptor 4; UA: Uric acid; XO: xanthine oxidase.

Inflammation mediated by the NLRP3 inflammasome is triggered by a pattern recognition receptor (PRR) after recognizing potentially harmful substances or signs of injury in the body [70]. Inflammation formation is mainly divided into two signaling phases [71]. Signal 1 is activated by the PRR and triggers the activation of a series of molecules, which ultimately activate NF-κB, which then enters the nucleus and binds to free DNA response elements, causing the transcription of target genes, including NLRP3 precursor, interleukin (IL)-1β, and IL-18 precursor [72,73]. Signal 2 is activated by a variety of pathogens and risk-associated factors, and the mode of activation is categorized as either pathogen-associated molecular patterns (PAMPs) or damage-associated molecular patterns (DAMPs) depending on the type of molecule activated [74]. After the activation of NLRP3 inflammatory vesicles by PAMPs or DAMPs, downstream caspase-1, IL-1β, IL-18, and gasdermin D (GSDMD, a perforating protein) are produced and released GSDMD N-terminally, resulting in an inflammatory effect as well as a mechanism of cellular pyroptosis, which severely damages cells and tissues [75,76]. Since MSU crystals are only involved in the second phase of the NLRP3 inflammatory pathway, this could largely explain the fact that not all hyperuricemia causes GA episodes in the clinic [6]. Studies have shown that ROS plays a very important role in the NF-κB-NLRP3 signaling pathway, both specifically activating NF-κB to cause the transcription of target genes and as a common second signaling molecule in the body, specifically activating the NLRP3 inflammatory vesicles, in which NIMA-related kinase 7 (NEK7) serves as a specific sensor for the ROS activation of NLRP3 inflammatory vesicles [77,78]. In addition, ROS can, likewise, activate NLRP3 inflammatory vesicles by promoting the dissociation of thioredoxin-interacting protein (TXNIP) from the antioxidant protein thioredoxin-1 (Trx1), which directly binds TXNIP to NLRP3-related structural domains [79,80,81]. Interestingly, the activated NLRP3 inflammasome can also play an immune role by recruiting neutrophils and macrophages by inducing an inflammatory response, from which ROS are released. Moreover, IL-1β, an effector inflammatory factor of NLRP3, promotes the accumulation of intracellular ROS by interfering with SOD and CAT, which has led to the speculation that a positive loop exists between ROS and the activation of the NLRP3 inflammasome [24,82]. Especially in the GA environment, the high concentration of MSU crystals is a co-activator of ROS and NLRP3 inflammatory vesicles, which puts the tissues of the organism in the same state of OS and inflammatory response and is more likely to cause the above-mentioned positive cycle of ROS-NLRP3 activation [83]. Thus, GA treatment needs to be combined with the two-pronged inhibition of NLRP3 inflammatory vesicles and ROS, which may play a significant role in the effect. Based on this theory, Luo et al. used Corilagin (a gallotannin with excellent anti-inflammatory and antioxidant effects) to intervene in the treatment of GA mice and found that the mice had reduced ROS production and inhibited inflammation specifically by unlinking TXNIP, NEK7, and NLRP3 inflammatory vesicles, which significantly ameliorated the GA mice’s symptoms, confirming that ROS-NLRP3 inflammation is an effective intervention pathway in GA [84]. The specific mechanisms are summarized in Figure 3.

### 4.2. ROS-MAPK

**Figure 4:**
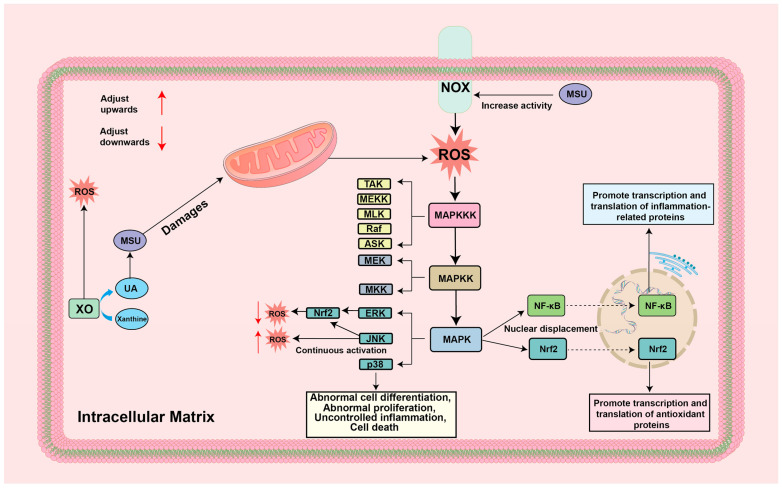
Mechanism of ROS-MAPK in GA environment: ASK/Raf/MLK/MEKK/TAK: A member of the MAPKKK family; ERK/JNK/p38: A member of the MAPK family; MAPK: mitogen-activated protein kinase; MAPKK: MAP kinase kinase; MAPKKK: MAP kinase kinase kinase; MEK/MKK: A member of the MAPKK family; MSU: monosodium urate; NF-κB: nuclear factor kappa-B; NOX: NADPH oxidase; Nrf2: Nuclear factor erythroid2-related factor 2; UA: Uric acid; XO: xanthine oxidase.

MAPK is a class of serine/threonine protein kinases located in cells, which can regulate the expression of intracellular genes through the MAPK cascade reaction, thus mediating life processes such as cell differentiation, proliferation, and apoptosis, making it a very important signaling network in cells [85,86]. The MAPK cascade reaction is mainly divided into three layers from top to bottom. The first two layers are MAP3K (TAK, MEKK, MLK, Raf, ASK, and others), and MAPK2K (MEK and MKK), which are also the prerequisite for the activation of MAPK [87,88]. MAPK, as a group of effector proteins, is located in the third layer of the cascade reaction, which mainly consists of extracellular signal-regulated kinase 1/2 (ERK1/2), c-Jun-N-terminal kinase 1–3 (JNK1–3), and p38MAPKα-δ (p38α-δ) [89]. It was found that ROS was active in various MAPK signaling pathways, and it was hypothesized that the activation of MAPK was regulated by ROS [90,91,92]. Further studies showed that the activation pathways of MAPK by ROS mainly included ROS/ras/RAF/MEK/ERK, ROS/ASK1/MKK4/JNK, and ROS/ras/MEKK/MLK3/ASK1/MKK/p38MAPK [93]. Among them, the activation of ERK prevents the damage caused by ROS and is involved in the process of cellular value-added differentiation. The activation of JNK can upregulate ARE-initiated transcription factors through Nrf2 phosphorylation to exert certain antioxidant effects, but it should be noted that the sustained activation of JNK can lead to apoptosis as well as the elevation of ROS [94,95]. p38MAPK activation can lead to abnormal cell differentiation, abnormal proliferation, uncontrolled inflammation, and even death [96,97]. However, studies at this stage have shown that the MAPK signaling pathway has a more pronounced activation of inflammation compared to the antioxidant effects exerted [98]. MAPK can mediate the phosphorylation of IκB kinase (IKK), an upstream molecule of NF-κB, as well as the degradation of the IκB family to promote the nuclear translocation of NF-κB. The unregulated MAPK signaling pathway can cause the impairment of a variety of cellular functions, thus leading to the development of a variety of diseases, including the activation of inflammatory and apoptotic pathways [99,100]. Numerous studies have shown that the effective reduction in paw and joint swelling and the improvement of inflammatory cell infiltration in tissues in GA mice can be achieved by the negative regulation of p38MAPK, JNK [101,102,103]. However, the paradox of the positive gains in antioxidant effects caused by the activation of MAPK signaling versus the various negative impacts, such as cellular inflammation, functional impairment, and other negative impacts, still needs to be verified and elucidated by subsequent scientific research. The specific mechanisms are summarized in Figure 4.

### 4.3. ROS-NET

**Figure 5:**
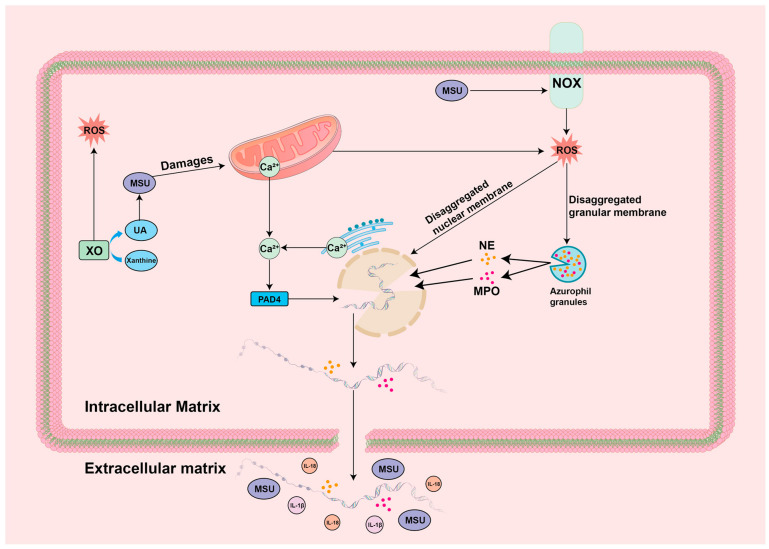
Mechanism of ROS-NET in GA environment: IL-1β/18: interleukin-1β/18; MPO: Myeloperoxidase; MSU: monosodium urate; NE: neutrophil elastase; NOX: NADPH oxidase; PAD4: peptidylarginine deiminase 4; ROS: reactive oxygen species; UA: Uric acid; XO: xanthine oxidase.

NET is an extracellular reticular DNA complex produced by neutrophils, which can be used as a substrate to trap foreign microorganisms or induce an immune response [104]. With the deeper study of NET, it was found that NETosis (the NET formation process) is induced by the nuclear depolymerization function of neutrophil elastase (NE) and myeloperoxidase (MPO), as well as the citrullination of histone arginine residues from peptidylarginine deiminase 4 (PAD4), where PAD4 is driven at high concentrations of Ca^2+^ [105]. In the above process, ROS are the dependent signaling molecules that drive the action of NE and MPO [106,107]. On the one hand, ROS can rupture and disassemble azurophil granules and nuclei by acting on azurophil granule membranes and nucleus membranes; on the other hand, ROS can promote the migration of NE from azurophil granules to the nucleus, degrade histones to aid chromatin decondensation, and promote MPO binding to chromatin to further enhance chromatin depolymerization, ultimately inducing NETosis [108,109,110]. Recent studies have shown that the ROS-induced DNA damage repair process also plays an important role in NETosis. In neutrophils induced to produce ROS, a large formation of 7,8-dihydro-8-oxoguanine (8-oxoG), one of the most common ROS-mediated DNA damage modification products, was observed, suggesting that ROS cause extensive DNA damage. During the subsequent process, large amounts of the DNA repair protein PCNA, a DNA clamp involved in the repair process, were observed to be heavily recruited into the nucleus DNA and were observed to be present throughout the NETosis DNA. At the same time, it was observed that inhibition of the initial chromatin decondensation step mediated by the BER/NER pathway significantly prevented NETosis, which is an early repair process of DNA damage, suggesting that the ROS-mediated DNA damage and repair process plays a significant role in NETosis [111]. It is also important to note that DNA transcription processes contribute equally to driving NOX-dependent or NOX-independent NETosis. It has been found that there is a high level of DNA transcription in the nucleus during NETosis and that transcriptional activity is significantly and positively correlated with the decondensation of chromatin required for NETosis. In contrast, a significant reduction in NET activation was observed by inhibiting transcription, and the transcription process did not affect ROS production in neutrophils [112]. Studies have shown that NET is a double-edged sword in the GA setting, and is closely associated with both the onset and remission of GA [113,114]. In the early stage of a GA attack, MSU crystals, on the one hand, can release large amounts of inflammatory factors to recruit neutrophils, and on the other hand, can lead to NETosis through the activation of the ROS-NET pathway. A variety of DAMPs is then released, which further induces a large amount of inflammatory mediators (e.g., tumor necrosis factor α/TNF-α, IL-6, and IL-8), thus exacerbating inflammation and leading to an acute GA episode [115,116]. At the peak of GA inflammation, NETosis can be followed by the formation of NET aggregates (aggNET), which have the ability to trap and degrade inflammatory mediators by relying on their intrinsic serine proteases, playing an important mitigating role during the GA inflammatory phase [117]. In addition, NETosis has also been shown to contribute significantly to the formation of tophus in the later stages of GA [118]. It is worth noting that aggNET was found to share highly similar features with gouty stones, including extracellularly dispersed MSU crystals with extended regions of DNA that are decorated with neutrophil granules. As mentioned above, aggNET can degrade inflammatory mediators, which happens to coincide with the frequent observation in clinical practice that signs that tophus in certain patients with chronic GA are in a long-term inflammatory resting state [119]. The specific mechanisms are summarized in Figure 5. 

### 4.4. ROS-Autophagy

**Figure 6:**
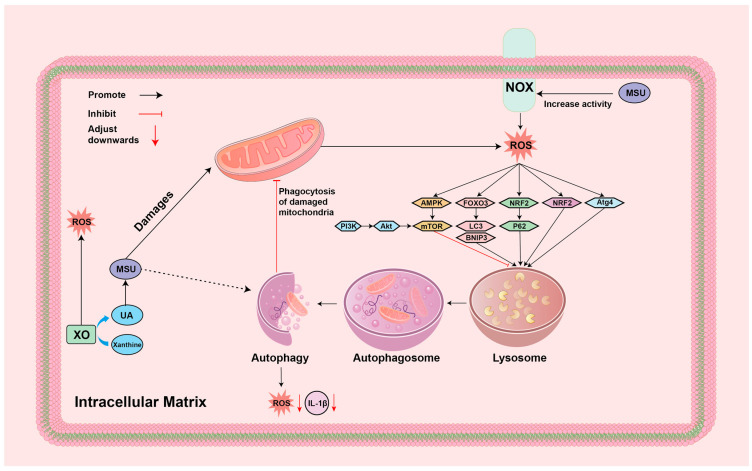
Mechanism of ROS-autophagy in GA environment: AKT: protein kinase B; AMPK: Adenosine 5′-monophosphate (AMP)-activated protein kinase; Atg4: Autophagy protein 4; BNIP3: BCL2/adenovirus E1B 19kDa interacting protein 3; FOXO3: Forkhead Box Protein O3; IL-1β: interleukin-1β; LC-3: microtubule-associated protein light chain 3; MSU: monosodium urate; mTOR: mammalian target of rapamycin; Nrf2: Nuclear factor erythroid2-related factor 2; P62: Sequestosome 1; PI3K: phosphoinositide 3-kinase; ROS: reactive oxygen species; UA: Uric acid; XO: xanthine oxidase.

Numerous studies have reported that the autophagy pathway is involved in the process of GA generation and mitigation, regulating inflammatory and metabolic responses [120,121,122]. The autophagy process is a metabolic process that utilizes lysosomes to degrade its own proteins and organelles, and a double-edged sword of the cellular metabolic process, which can protect the cell by breaking down harmful substances, but may also lead to cellular damage or even death [123,124]. Autophagy has multiple activation mechanisms, and one of the more important activation pathways is mediated by ROS, which includes a variety of molecular signaling pathways such as ROS-FOXO3-LC3/BNIP3, ROS-NRF2-P62, ROS-AMPK, ROS-ATM, and ROS-Atg4 autophagy [125,126,127]. Interestingly, the activation of ROS autophagy can in turn contribute to the reduction in ROS levels, and further studies have shown that this is related to the process of mitochondrial autophagy that occurs in damaged mitochondria as well as the removal of oxidized proteins and blocking of ROS production [128,129,130]. In addition, another class of negatively regulated autophagy pathways exists, where the inhibition of PI3K-AKT mammalian target of rapamycin (mTOR) signaling activation promotes autophagy and, thus, reduces ROS production [131,132,133]. Hsieh and his team used an autophagy activator to intervene in a model of GA and concluded that the activation of autophagy significantly alleviated oxidative stress and inflammation in GA tissues. This is due to the ability of autophagic processes to phagocytose and degrade damaged mitochondria to prevent the release of ROS, and the ability to degrade inflammatory factors, thus suggesting that intervening in autophagy could be an effective means of treating GA [134]. The specific mechanisms are summarized in Figure 6.

### 4.5. ROS-Ferroptosis

**Figure 7:**
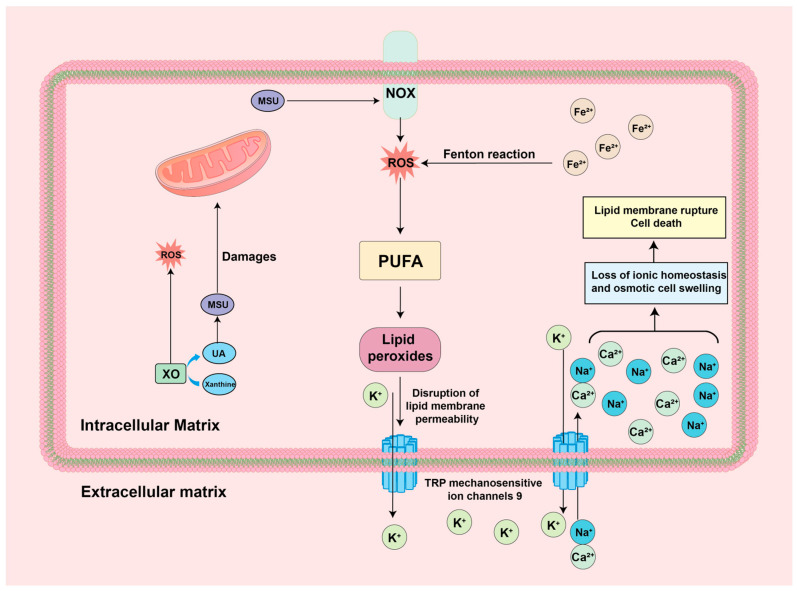
Mechanism of ROS-Ferroptosis in GA environment: MSU: monosodium urate; NOX: NADPH oxidase; PUFA: polyunsaturated fatty acid; ROS: reactive oxygen species; TRP: transient receptor potential; UA: Uric acid; XO: xanthine oxidase.

Investigative studies have found that Ferroptosis, as a part of the oxidative stress process, is also strongly associated with the course of GA [135]. Ferroptosis is a process in which intracellular free iron or iron-containing enzymes react with ROS and polyunsaturated fatty acid (PUFA)-containing lipids to produce high levels of membrane lipid peroxides and subsequently promote cell death [136]. It has been shown that the formation of lipid peroxides is related to intracellular iron ion concentration, PUFA content, and intracellular ROS content [137,138]. When the suitable conditions are met, a large number of lipid peroxides are formed in the cell, which act on the cell membrane and lead to the disturbance of ion channels, further leading to the loss of ion homeostasis and osmotic cell swelling, and, ultimately, plasma membrane rupture and cell death [139,140]. Serum levels of ferritin and transferrin were positively associated with the risk of hyperuricemia and the number of episodes of GA, as reported in a national study in China [141]. Further studies have suggested that Fe^2+^ may be involved in Ferroptosis with the GA process by promoting the XO expression and activation process to promote UA and ROS generation [142]. It has also been found that long non-coding RNA (lncRNA) ZNF883, a key gene associated with Ferroptosis production, is significantly downregulated in GA and plays a role in gout patients by regulating PRKAA1 (sequence of a key gene for adenosine monophosphate-activated protein kinase) [143]. Li et al. demonstrated that Ferroptosis was observed in cells treated with high UA, and after the application of Fer-1 (an iron death inhibitor), the oxidative status and UA levels in the cells were significantly reduced, suggesting that a palliative effect on the course of the disease can be achieved by intervening with Ferroptosis in high-UA-induced diseases. Unfortunately, there is no direct evidence of Ferroptosis intervention for GA [144]. The specific mechanisms are summarized in Figure 7.

### 4.6. ROS-Nrf2 Antioxidant Response

**Figure 8:**
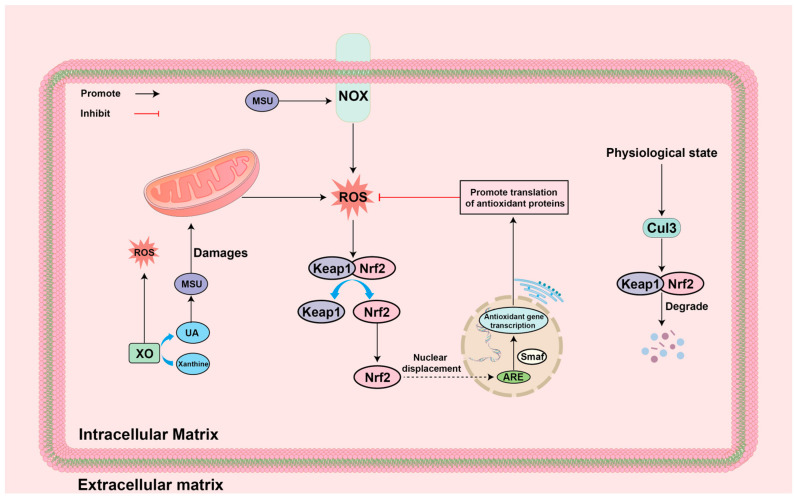
Mechanism of ROS-Nrf2 antioxidant response in GA environment: Cul3: Cullin 3; Keap1: Kelch-like ECH-associated protein 1; MSU: monosodium urate; NOX: NADPH oxidase; Nrf2: Nuclear factor erythroid2-related factor 2; ROS: reactive oxygen species; UA: Uric acid; XO: xanthine oxidase.

Nrf2, a transcription factor identified to regulate beta-globin gene expression, is involved in the positive regulation of Antioxidant Response Element (ARE) in human RNA and drives the expression of antioxidant enzymes [145]. Under physiological conditions, Nrf2 binds to Kelch-like ECH-associated protein 1 (Keap1), which inhibits Nrf2 nuclear translocation and contributes to the ubiquitination (ubiquitinated) of Nrf2 in the presence of Cullin 3 (Cul3) E3ubiquitin ligase, and eventually is degraded by the proteasome [146,147]. However, in the OS state, cysteine residue located in Keap1 are oxidatively modified, mediating a conformational change in Keap1, which prevents Nrf2 ubiquitination and increases the stability of Nrf2, causing it to accumulate and translocate to the nucleus. In the nucleus, Nrf2 can heterodimerize with small musculoaponeurotic fibrosarcoma (sMaf) protein and binding to ARE, which induces the expression of numerous antioxidant defense proteins, including NADPH, GSH, GPX, PRX, TRX, etc., and exerts antioxidant effects to advantageously repair the oxidative internal environment in cells [148,149]. Recently, it has been shown that activation of Nrf2 antioxidant signaling significantly alleviated mechanical abnormal pain and ankle edema and improved gait impairment in GA model mice, reduced inflammatory cell infiltration in ankle tissues, and decreased ROS production induced by MSU crystals as well as the expression of ROS-induced related inflammatory factors [150,151]. The specific mechanisms are summarized in Figure 8.

## 5. Targeted ROS Network Drug Therapy for GA

As mentioned earlier, ROS, as effector and signaling molecules in the GA environment, not only cause damage to proteins, cells, and tissues by OS, but also regulate processes such as inflammation, cellular pyroptosis, NET, and Ferroptosis in the body to damage joint tissues. Therefore, targeting and regulating signaling molecules in the ROS network is expected to block pathological processes such as OS, inflammation, and NET, ameliorate tissue damage in GA, alleviate the symptoms of GA, and has great potential to inhibit the formation of tophus.

### 5.1. Targeting ROS-NLRP3 for the Treatment of GA

ROS-NLRP3 inflammation occupies a very important role in the onset of GA. Several ROS-NLRP3 blocking agents were tested in pre-clinical GA models. Recent studies show that the phenolic monoterpene carvacrol has anti-inflammatory and antioxidant pharmacological effects [152]. Muhammad et al. reported that carvacrol significantly downregulated uric acid levels in GA model mice, reduced oxidative stress by activating endogenous antioxidant proteins, and blocked the activation of ROS-NLRP3 inflammation, which resulted in a better joint protective effect [153]. Rutin, another natural flavonoid compound, is widely found in various plants such as tomatoes, carrots, sweet potatoes, and apple peels, and has a good inhibitory effect on neutrophil infiltration and oxidative stress [154]. Wu and colleagues showed that Rutin was also effective in the treatment of GA, exerting anti-inflammatory effects by inhibiting ROS production, restoring oxidative stress homeostasis, and inhibiting ROS-NLRP3 inflammatory activation, as well as ameliorating the degree of joint swelling in the quails’ GA model [155]. In addition to the therapeutic role of drugs in targeting the ROS-NLRP3 pathway, acupuncture also has significant therapeutic effects. Acupuncture, an ancient Chinese medicine technique, is used to treat a wide range of diseases because of its safety, low risk, potent treatment, and low cost [156]. With the advancement of modern medical technology, acupuncture combined with electrophysiological effects has evolved into the form of Electroacupuncture (EA), which not only combines the advantages of the traditional acupuncture mentioned above, but also has the advantage of controlling and quantifying the intensity and frequency of acupoint stimulation [157]. Wei’s team reported that electroacupuncture of Zusanli (ST36) and Kunlun (BL60) acupoints in a GA mouse model downregulated the production of ROS and NLRP3 inflammatory vesicles, significantly attenuated neutrophil infiltration in the synovial membranes of ankle joints of mice with a gout model, and reduced TRPV1 channels in neurons, which attenuated nociception in mice [158].

### 5.2. Targeting ROS-MAPK for the Treatment of GA

MAPK plays a role in regulating the production of inflammatory cytokines and triggering aberrant changes in synoviocyte signaling, leading to apoptosis and proliferation of synoviocytes and causing severe damage to joint tissues [159]. At this stage, studies have shown that interfering with ROS-MAPK can effectively ameliorate the inflammatory response and tissue damage in GA [86]. A study reported that Cangzhu, a widely used traditional Chinese medicine, could improve inflammation, reduce synovial cell proliferation, and alleviate joint tissue cell dysregulation by inhibiting XOD levels and the expression of MAPK and NF-κB in the joint tissues of mice with GA [160]. In addition, Mastoparan-M, one of the main active components of wasp venom, has been shown to inhibit ROS generation, downregulate the expression of P38, JNK, and NF-κB, and further block the inflammatory response and apoptosis guided by MAPK and NLRP3, thus exerting its powerful antioxidant and anti-inflammatory effects and reducing the swelling of GA joint tissues [161].

### 5.3. Targeting ROS-NET for the Treatment of GA

On the one hand, NET activation releases reticular contents including DNA and various granule proteins, which can act as DAMPs to trigger the inflammatory cascade response, which plays an important contribution in the early stage of inflammation in GA [162]. Since NET activation is inextricably linked to ROS, targeted ROS-NET intervention may be a potential therapeutic target for GA [163]. Zhou et al. found that Dioscorea nipponica Makino, a natural drug, significantly reduced key components of NET in joint tissues of GA mice, including the downregulation of NE, PR3, CTSG, LTF, and MPO, and played the anti-inflammatory effect by decreasing the expression of IL-1β and TNF-α [164]. Furthermore, Suzuki et al. demonstrated that the presence of NET not only caused inflammation but also played a crucial role in prolonging and intensifying local inflammation and pain, whereas treatment with deoxyribonuclease degraded the composition of NET and significantly alleviated muscle joint pain sensitivity in GA mice [165]. Jia’s team also suggested that the occurrence of NET inhibits the viability of osteoblasts and enhances the activity of osteoclasts in the joint tissues of GA mice, resulting in severe bone erosion, and that blocking the formation of ROS-NET may be effective in alleviating the damage of GA joints [111]. On the other hand, the formation of NET may also serve as the material basis of gouty stones, and intervening in the formation of NET may be effective in preventing the formation of gouty stones [166]. Regrettably, however, although there is some basic research and theoretical support, the corresponding drug development has not been able to carry out basic research. Although it has been reported in recent years that Pegloticase can effectively improve the size of gout stones, the specific mechanism, whether it is related to the blockage of ROS-NET, and whether it has a preventive effect on the incidence of tophus, are not yet known [167].

### 5.4. Targeting ROS-Autophagy for the Treatment of GA

Recent studies have shown that some natural products can regulate autophagy and thus alleviate GA inflammation and oxidative stress. 3β,23-Dihydroxy-12-ene-28-ursolic Acid, an active triterpenoid isolated from *C. paliurus*, was found to promote autophagy and eliminate oxidative stress and inflammation levels in GA tissues through autophagy by decreasing the level of ROS, increasing the expression of autophagy marker LC3, and blocking the PI3K-AKT-mTOR signaling pathway, thus promoting autophagy and eliminating oxidative stress and inflammation levels in GA tissues through autophagy [168]. Chinese medicine prescription is a traditional treatment applied for thousands of years. Han et al. applied Zisheng Shenqi Decoction to intervene in GA mice and found that it could upregulate the levels of AMPK, Beclin-1 (a key initiator of autophagy), microtubule-associated protein 1 light chain 3 II/I (LC3II/I), SOD, glutathione peroxidase (GSH-Px), and CAT to activate autophagy and antioxidants. And it could reduce the expression of NLRP3 and its related inflammatory factors by regulating autophagy, which significantly improved the inflammatory cell infiltration in GA and reduced the swelling of joints in mice [169].

### 5.5. Targeting ROS-Ferroptosis for the Treatment of GA

The current study showed that serum ferritin and transferrin levels were abnormally elevated in GA patients and positively correlated with the number of GA episodes. MDA, a marker of Ferroptosis, was significantly elevated in GA tissues, which, in combination with the long-term oxidative stress in the GA joints, suggests that Ferroptosis is highly likely to occur in the GA joints [141,170]. Shao and his team aptly confirmed the above by the identification of lncRNAs, and found that the key gene for Ferroptosis, lncRNA ZNF883, was significantly downregulated during GA, suggesting that Ferroptosis was active in GA tissues [143]. This suggests that ROS-Ferroptosis is another key target for GA treatment. However, the current study of anti-Ferroptosis drugs has not been carried out in GA, and it is still necessary for colleagues to intensify their research efforts so that potential ROS-Ferroptosis targets can be verified and efficient drugs with an anti-Ferroptosis mechanism can be developed as soon as possible.

### 5.6. Targeting ROS-Nrf2 for the Treatment of GA

As mentioned earlier, ROS contribute significantly to the course of GA, and control of the production of ROS itself is particularly important in the treatment of GA. The major ROS elimination pathway is mediated by regulating the ROS-Nrf2 pathway to produce a variety of antioxidant proteins, and the promotion of ROS-Nrf2 activation has been an important target for antioxidant effects [171]. After Zeng et al. applied oltipraz (an Nrf2 activator) to GA mice, they found that oltipraz significantly alleviated MSU-induced ROS production and inflammation, reduced the function of pain-sensing TRPV1 channels in DRG neurons, and improved ankle gait disturbance and inflammatory cell infiltration in mice [172]. Artemisia selengensis Turcz is a traditional medicinal plant used for the treatment of many diseases, with biological activities such as antioxidant, antimicrobial, anticancer, anti-inflammatory, anti-malarial and anti-hyperglycemic effects [173]. Cao et al. found that Artemisia selengensis Turcz also had significant therapeutic effects in GA by promoting nuclear translocation of Nrf2, reducing MUS-induced ROS production, and decreasing NLRP3 protein expression as well as the release of inflammatory factors, thereby alleviating oxidative stress and inflammation in GA [174].

Specific study data are summarized in Table 1:

## 6. Conclusions and Perspectives

The living standards of human beings are continually increasing, and the incidence of GA, which is a “disease of affluence”, has also risen. GA usually occurs in combination with diabetes mellitus, hypertension, obesity, chronic kidney disease, and cardiovascular and other chronic diseases, and is accompanied by a high rate of disability, seriously jeopardizing the quality of life of patients [200]. Regarding the treatment of acute exacerbations of GA, the current clinical first-line drugs are divided into three main categories: non-steroidal anti-inflammatory drugs (NSAIDs), colchicine, and glucocorticoids, which can provide rapid anti-inflammatory and analgesic effects, and shortening the disease process [201,202]. However, the three major mainstream classes of drugs have numerous adverse effects and limitations; NSAIDs can cause gastrointestinal problems ranging from mild dyspepsia to gastrointestinal perforation, ulcers, and bleeding, and chronic renal insufficiency may result from the long-term use of higher doses. Colchicine users also suffer from gastrointestinal side effects including nausea, vomiting, diarrhea, cramps, and pain, as well as occasional headache and fatigue. In addition, colchicine is contraindicated in patients with hepatic or renal injury. The side effects of glucocorticoid use include hyperglycemia, infections, gastrointestinal bleeding, water and sodium retention, osteoporosis, and many other adverse effects, significantly limiting its use; moreover, GA is still prone to recurring despite treatment with all three classes of drugs [203,204,205,206]. In this context, it is particularly urgent to understand the micro-mechanisms underlying GA attacks and to identify more efficient and safe treatment options.

Current studies have shown that the micropathologic mechanism of gout is closely related to NLRP3 inflammation, OS, NET, autophagy, Ferroptosis, etc. However, after synthesizing the above mechanisms, it can be found that ROS are present almost throughout the whole process of gout onset, progression, remission, and tophus formation, jointly forming a huge network of multiple mechanisms involved in the GA disease process. Firstly, ROS can act as an effector molecule of OS, and the accumulation of a large amount of ROS can directly attack the cell membrane, protein, and DNA of the joint tissue cells, leading to the destruction of cell function or even cell death [207,208]. Secondly, ROS can act as a signaling molecule involved in the activation of NLRP3 inflammation, NET, MAPK, autophagy, and Ferroptosis, which can indirectly cause serious damage to the joint tissues [209,210,211,212,213]. Meanwhile, numerous reports collected in this review also showed that studies that used substances with antioxidant effects to intervene in GA mice achieved significant therapeutic effects, significantly improving the inflammation and OS status of GA, protecting joint tissue cells, alleviating pain, and reducing the pace of pathology in GA mice, as well as preventing the formation of tophus. This suggests that the development of composite target drugs for the treatment of GA using signals in the ROS network is expected to block the process of GA from the fundamental mechanism and break through the limitations of the current treatment, showing considerable potential for development and clinical application.

However, due to the fact that the mechanism and role of ROS have not been fully elucidated in the current GA research field, a number of challenges and unresolved problem remain. (1) The depth of research is limited: The description of the specific mechanism in terms of how ROS are involved in the formation of GA is still imperfect, and most studies are only based on ROS as an indicator. However, the roles of upstream and downstream targets involved in ROS have not been deeply explored, resulting in ambiguity in the understanding of ROS’s contribution to the formation of GA, which should be addressed in subsequent studies. (2) Limitations of research efforts: The current experimental samples of GA treatment using ROS network targets are relatively small, and although there is considerable efficacy, the results are still unconvincing. In particular, the ROS-autophagy, ROS-NET, and ROS-Ferroptosis pathways are still at the theoretical level, and their therapeutic effects on GA are still to be proven by subsequent researchers. (3) Limitations of the research process: The current research on the use of ROS network targets for the treatment of GA is still at the level of basic animal experiments, and there is a lack of strong data from large-sample, multi-center, randomized, double-blind clinical trials, meaning that we are a long way from the development of drugs that can be used in clinical treatment.

In addition, this paper discusses the mechanisms related to the ROS network and the development of GA, especially the generation, mechanism of action, and metabolic pathway of ROS. In this process, we found that the signaling pathways involved in GA include OS, NLRP3 inflammation, the Nrf2 pathway, autophagy, the MAPK pathway, and the NET pathway. These are not single independent processes, and they contain multiple complex and closely linked systems such as tandem, cyclic, and crossover processes. It has been reported that MSU crystals significantly activate the expression of ROS and NLRP3 inflammatory vesicles in a high-uric-acid environment, and that drugs activated by inducing autophagy can effectively block ROS-NLRP3-mediated inflammatory injury [214,215]. Moreover, MSU also induces the formation of neutrophil autophagy, which can then be involved in NETosis and reduces the expression of inflammatory factors such as IL-1β, IL-6, TNF, and MCP-1 to alleviate the acute inflammatory response to GA episodes [216,217]. There are also studies that show that neutrophil autophagy generates certain ROS, which bear the intermediate signaling link between neutrophil autophagy and NET formation [218]. ROS undergo an immune effect after the activation of NLRP3 inflammation, causing neutrophil aggregation, which further promotes ROS production [219]. It has also been shown that activation of ROS-MAPK induces IKK phosphorylation and the degradation of the IκB family, which, in turn, induces the activation of NLRP3 inflammatory vesicles and the cross-activation of ROS-MAPK-Nrf2 and ROS-MAPK-NLRP3 inflammation [94].

The above findings suggest that a vast network of mechanisms centered on ROS covers the major micro-mechanisms of GA, which are complex and tightly interconnected. A future direction of research could involve using ROS as the central point, to investigate the joint ROS networks of multiple targets and pathways so as to assist in the development of therapeutic drugs for GA. This way, interventions can be developed that use the micro-mechanisms from multiple directions, blocking the process of GA production. This method can not only avoid the current status quo of treating the symptoms but not the root cause, but would also have a more comprehensive effect and a wider coverage mechanism compared with single-target drugs, thus improving upon the existing treatment methods.

Our team has reason to believe that the development of anti-GA drugs by targeting the ROS network will make satisfactory progress in the near future.

**Figure 9:**
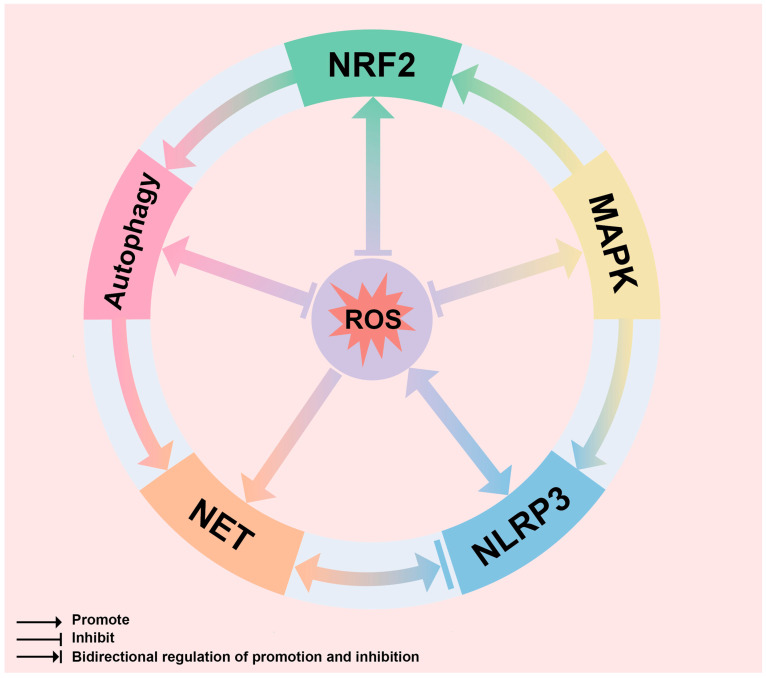
Diagram of ROS network crosstalk mechanism: NRF2: Nuclear factor erythroid2-related factor 2; ROS: reactive oxygen species; MAPK: mitogen-activated protein kinase; NLRP3: NOD-like receptor thermal protein domain-associated protein 3; NET: neutrophil extracellular traps.

The ROS network crosstalk relationship is shown in Figure 9.

## Figures and Tables

**Table 1:** Summary of therapeutic effects of drugs targeting the ROS network in GA conditions.

Therapeutic Target	Mechanisms	Therapeutic Effect	Bibliography
ROS-NLRP3	ROS activate NLRP3 inflammatory vesicles, release inflammatory factors, and in turn induce ROS production	Reduces ROS production, attenuates the association between ROS and NLRP3, disrupts the vicious cycle of oxidation and inflammation, and relieves inflammation	[175,176,177,178,179,180,181,182,183,184,185,186,187]
ROS-MAPK	ROS activate multiple pathways in the MAPK cascade to further activate MAPK, ultimately inducing inflammation and apoptosis	Reduces ROS production and disrupts the upstream activation pathway of MAPK to alleviate cellular inflammation and apoptosis	[160,188,189,190]
ROS-NET	ROS induce NETosis, promote cell rupture, release DAMPs, and trigger inflammatory effects. Massive release of reticulocyte contents, as the material basis of tophus	Reduces ROS production, prevents NETosis from upstream mechanisms, relieves inflammatory response, and prevents tophus formation	[191]
ROS-autophagy	ROS activate autophagy upstream via multiple pathways, and autophagy further reduces ROS and inflammation by phagocytosis of damaged mitochondria and other organelles	Reduces ROS production and promotes autophagosome formation to further alleviate oxidative stress and inflammation formation	[134,192,193]
ROS-Ferroptosis	Intracellular free iron or iron-containing enzymes react with the lipids of PUFA and ROS to produce high levels of lipid peroxides, thereby disrupting the biophysical properties of the plasma membrane leading to rupture of the cell membrane and cell death	Inhibits the production of ROS, reduces the formation of lipid peroxides, and prevents the rupture of cell membranes due to peroxidation, leading to cell death	No treatment has been reported.
ROS-Nrf2	ROS activation upstream signaling promotes Nrf2 nuclear translocation, the formation of multiple antioxidant proteins, and the subsequent degradation of excess intracellular ROS	Inhibition of ROS production, activation of Nrf2, promotion of nuclear translocation, and further degradation of ROS	[194,195,196,197,198,199]
